# Tissue Engineered Materials in Cardiovascular Surgery: The Surgeon's Perspective

**DOI:** 10.3389/fcvm.2020.00055

**Published:** 2020-04-15

**Authors:** Andras P. Durko, Magdi H. Yacoub, Jolanda Kluin

**Affiliations:** ^1^Department of Cardiothoracic Surgery, Erasmus University Medical Center, Rotterdam, Netherlands; ^2^Imperial College London, National Heart and Lung Institute, London, United Kingdom; ^3^Department of Cardiothoracic Surgery, Amsterdam University Medical Center, Amsterdam, Netherlands

**Keywords:** tissue-engineering, bioengineering, cardiac surgery, heart surgery, *in-situ* tissue engineered, TEHV

## Abstract

In cardiovascular surgery, reconstruction and replacement of cardiac and vascular structures are routinely performed. Prosthetic or biological materials traditionally used for this purpose cannot be considered ideal substitutes as they have limited durability and no growth or regeneration potential. Tissue engineering aims to create materials having normal tissue function including capacity for growth and self-repair. These advanced materials can potentially overcome the shortcomings of conventionally used materials, and, if successfully passing all phases of product development, they might provide a better option for both the pediatric and adult patient population requiring cardiovascular interventions. This short review article overviews the most important cardiovascular pathologies where tissue engineered materials could be used, briefly summarizes the main directions of development of these materials, and discusses the hurdles in their clinical translation. At its beginnings in the 1980s, tissue engineering (TE) was defined as “*an interdisciplinary field that applies the principles of engineering and the life sciences toward the development of biological substitutes that restore, maintain, or improve tissue function”* (1). Currently, the utility of TE products and materials are being investigated in several fields of human medicine, ranging from orthopedics to cardiovascular surgery (2–5). In cardiovascular surgery, reconstruction and replacement of cardiac and vascular structures are routinely performed. Considering the shortcomings of traditionally used materials, the need for advanced materials that can “restore, maintain or improve tissue function” are evident. Tissue engineered substitutes, having growth and regenerative capacity, could fundamentally change the specialty (6). This article overviews the most important cardiovascular pathologies where TE materials could be used, briefly summarizes the main directions of development of TE materials along with their advantages and shortcomings, and discusses the hurdles in their clinical translation.

## Clinical Need For Advanced Materials In Cardiovascular Surgery

### Congenital Heart Disease

Congenital heart defects affect ~9 of 1,000 newborns (7) and often require corrective surgery at an early age. Invasive treatment of congenital cardiac defects results in an increased life expectancy and can significantly improve quality-of-life (8).

Repair with a prosthetic patch is the cornerstone of reconstruction of diseased or defective cardiac and vascular structures in pediatric cardiac surgery. Patches are used for the closure of atrial or ventricular septal defects, for complex reconstructions in atrioventricular canal defects; in right ventricular outflow tract reconstruction in Tetralogy of Fallot; for aortic reconstruction in interrupted aortic arch or hypoplastic left heart syndrome; or when establishing cavo-pulmonary connection is required (9–13). As most of these operations are performed at very young or even neonatal age, repair must stay effective and durable in a rapidly changing physiological environment. Traditionally used materials—autologous or xenogeneic pericardium, or prosthetic materials like Dacron (DuPont, Wilmington, DE) or PTFE (polytetrafluoroethylene)—are suboptimal in this respect as they tend to calcify over time and cannot grow with the child (14–16).

As children will inevitably outgrow a prosthetic heart valve (PHV) implanted at young age, reconstruction of native valves is always preferred over replacement whenever feasible. However, if reconstruction fails or appears not possible, replacement of the dysfunctional valve with a PHV becomes necessary. In childhood, most commonly the pulmonary valve requires replacement, often using a right ventricle-to-pulmonary artery conduit (17). Unfortunately, none of the currently available allogenic or xenogeneic conduits can be considered ideal, as they cannot grow and degenerate on the long term (18–21). Besides pathologies of the pulmonary valve and right ventricular outflow tract, congenital aortic stenosis (AS) often necessitates valve replacement in the pediatric population. Although balloon palliation can buy some time until valve repair or replacement (22), patients with congenital AS often require a sequence of re-operations until they reach adulthood (23, 24), largely due to the absence of an optimal valve substitute.

### Acquired Valvular Heart Disease

Parallel to aging of the adult population, degenerative valvular heart diseases are becoming increasingly prevalent in the western world (25). Additionally, in developing countries, rheumatic valvular heart disease causes a substantial and often underestimated burden (26). Although valve replacement with a PHV improves symptom status and long-term survival, currently available heart valve prostheses are also associated with certain complications (27, 28). Mechanical PHVs necessitate lifelong anticoagulation, which increases the risk of bleeding and thromboembolic events and is suboptimal for women in childbearing age (29, 30), while the limited long-term durability and inherent structural degeneration of bioprosthetic valves remain a major issue for the younger patient population (31, 32).

### Vascular Grafts

In coronary artery bypass grafting, peripheral vascular reconstructions or when creating arteriovenous shunts for renal dialysis, small caliber vascular grafts are required. Although autologous vessels harvested from other parts of body are potentially ideal for this purpose, they are not always available or eligible for use. Traditionally used prosthetic grafts have numerous limitations due to their limited patency (33–35) and increased susceptibility for infections (36). This, together with the magnitude of the affected population necessitates intensive research for alternative solutions (37).

## Overview Of Tissue Engineered Solutions

Tissue engineering, by providing advanced materials with physiological function and ability for growth and regeneration, could potentially fulfill these clinical needs. To create a TE product, the following are required: (i) a (biodegradable) scaffold to guide tissue formation; (ii) cells able to populate the scaffold; and (iii) (in the classical way of TE) a bioreactor, which simulates a physiological environment to augment tissue formation. Scaffolds used in cardiovascular TE can be from various origin. The most commonly used scaffold materials are summarized in (Table 1). Similarly, multiple cell-types might be used: among others, mesenchymal stem cells, endothelial progenitor cells or induced pluripotent stem cells can be utilized during the TE process (56). Bioreactors are specifically designed containers intended to provide an optimized, controlled environment where cell-scaffold interaction can take place (57). During “classical” TE, scaffolds are seeded with progenitor cells and incubated *in vitro* in a bioreactor. Following a period of maturation under controlled conditions, the TE product is implanted to the patient. Besides this “conventional” approach, various other, “incomplete” methods exist, where one or more steps of the “conventional” TE process are bypassed (58).

**Table 1:** Most commonly used scaffold types in cardiovascular tissue engineering with examples.

**Scaffold type**	**Examples**			
	**Implant type**	**Implant position**	**Species**	**Reference**
Biological origin				
Decellularized vessels or valves				
Allogenic	Aortic root	Aortic	Human	(38)
Xenogeneic	Aortic root	Pulmonary	Ovine	(39)
Acellular or decellularized other xenogeneic tissues				
Pericardium	Patch	Various	Human	(40)
Small intestinal submucosa	Patch	Intracardiac	Human	(41)
	Valve	Tricuspid	Ovine	(42)
	Patch	Aortic arch	Human	(43)
	Patch	Right ventricular wall	Ovine	(44)
Allogenic or autologous engineered tissue				
	Valved conduit	Pulmonary	Canine	(45)
	Patch	Pulmonary	Human	(46)
	Vascular graft	A-V shunt	Human	(47–49)
	Valve	Aortic	Ovine	(50)
Synthetic origin				
	Valved conduit	Pulmonary	Ovine	(51, 52)
	Valve	Aortic	Ovine	(53)
	Vascular graft	Cavo-pulmonary connection	Human	(54)
	Valve	Pulmonary	Ovine	(55)

Some “TE” products are already cleared for clinical use: decellularized valves, porcine small intestinal submucosa and decellularized bovine pericardium (SynerGraft®, CryoLife Inc, Kennesaw, GA, United States; CorMatrix®, CorMatrix Cardiovascular, Roswell, GA, United States; CardioCel® Bioscaffold, LeMaitre Vascular, Burlington, MA, United States) are already parts of cardiovascular surgeon's armamentarium. Apart from decellularized allografts and porcine small intestinal submucosa, all other products are treated with glutaraldehyde (59) which can have a negative impact on cellular ingrowth following implantation (60). Furthermore, it is unclear if the above mentioned decellularized scaffolds degrade at all and whether they can truly be considered as TE products. Besides, all these products have inherent shortcomings. Allogenic tissues are generally cumbersome to procure and might still fail in the long term (61). Xenogeneic tissues can provoke inflammatory response leading to early degeneration and calcification. The first commercially available decellularized porcine valve dramatically failed due to a strong inflammatory response resulting in rapid degeneration and early structural failure in pediatric patients (62). Although a promising concept, acellular porcine small intestinal submucosa patches can also provoke an inflammatory response and can demonstrate early degeneration or calcifications leading to valve insufficiency when used for aortic valve repair (63, 64), or aneurysm formation when used for aortic reconstruction in pediatric patients (65). Decellularized bovine pericardium, though treated with low concentrations of glutaraldehyde (66), was found to be safe and effective in the mid-term when used for patch repair of complex congenital cardiac anomalies (40) and exhibited greater strain resistance compared to porcine small intestinal submucosa (67). Nevertheless, the possibility of calcification of the decellularized bovine pericardium has also been raised recently (68) and the quest for the “ideal” tissue engineered material continues.

## *In Situ* Tissue Engineering With Polymer Scaffolds

During *in situ* TE, an unseeded biodegradable scaffold is implanted to the recipient. After implantation, the scaffold will be populated *in vivo* by cells scrambled from the circulation, with the recipient's own body acting as a bioreactor. This simplified approach saves substantial costs and prevents potential complications associated with incubating the scaffold in a bioreactor. Neo-tissue formation begins only after scaffold implantation and can occur under completely physiological shear and pressure conditions (69).

Compared to biological materials, polymers materials are relatively easy to produce, handle, sterilize or store, and they can be manufactured in virtually any size or form. This creates the possibility of manufacturing directly off-the-shelf available TE cardiovascular implants and increases the interest in *in situ* TE using polymer scaffolds.

However, this technique also has certain shortcomings which have to be considered. During *in situ* TE, neo-tissue formation occurs in a less-controlled environment and cells repopulating the scaffold must be gathered from high blood flows which might not be ideal (70). Furthermore, a delicate balance between the pace of scaffold degeneration and neo-tissue formation is required to achieve an optimal result. Fortunately, in contrast to TE products from biological origin, the design of the prosthesis and the characteristics of the polymer material such as scaffold composition, scaffold or fiber thickness or fiber orientation can be relatively easily modified, if necessary (71–75).

Heart valve constructs from polymer scaffolds are currently under investigation in preclinical experiments. In sheep, these scaffolds have demonstrated satisfactory durability and function on mid- term when implanted in the pulmonary position as a surgical prosthesis (55) or as a valved conduit (76), or when used as a transcatheter aortic valve (53). Based on these encouraging results, the first-in-man investigations of the polymer scaffolds has recently been started (54).

## Challenges In Clinical Translation

As any new technology awaiting clinical introduction (77), TE products in cardiovascular surgery must find a therapeutic gap, a “niche,” where no ideal treatment option exists, and where the advantages of the new technology can be proven. Although the shortcomings of currently used materials are evident and some “TE” products are already approved and used in the clinical setting, there are many hurdles to overcome before TE materials can be routinely used in cardiovascular surgery (78).

Cardiovascular implants have to fulfill strict safety and performance criteria during their regulatory assessment before clinical introduction (79–81). During regulatory assessment, standards of the International Organization for Standardization (ISO) are widely used. The ISO 5840 standard on heart valve prostheses provides guidance on *in vitro* and *in vivo* hemodynamic and durability testing, as well as Objective Performance Criteria (OPC) for the assessment of complications after implantation (82). However, regulatory assessment of TE implants is complicated. During manufacturing, achieving consistence in the biological properties of cell-based products between batches is difficult, rendering TE products less reproducible than conventional cardiovascular implants. Furthermore, difficulties in sterilization, packaging and storage of cell-based TE products further limit their regulatory approval and widespread clinical use. On the other hand, *in situ* TE cardiovascular implants cannot be considered as “final products,” as their properties are expected to change after implantation while they transform into normally functioning living tissue. Of note, this transformation might not be the same in all subjects receiving the same implant and tissue formation might occur differently in animals used for pre-clinical *in vivo* testing than in human recipients (83). These issues make the interpretation of the results derived from *in vitro* and *in vivo* testing cumbersome, and together with the plethora of approaches and techniques used for manufacturing, makes the regulatory assessment of TE cardiovascular implants difficult. To date, no specific ISO standard on TE heart valves exist and it is not clear how these products should be classified or assessed.

Besides, considering these unique properties of TE cardiovascular implants, important ethical issues might arise when it comes to *in human* testing or clinical introduction of these products (84). Irrespective of the local circumstances or the clinical need, the risk of implanting a prosthesis that might fail must always be carefully weighed against the perceived benefits (26, 85, 86).

Another important aspect of successful clinical introduction is the cost-effectiveness of the novel device or technique, compared to standard treatment (87). Although development of TE materials are expensive, *in-situ* TE valves constructed from biodegradable polymer scaffolds could be potentially cost effective, according to a recent early health technology assessment study (88).

## Perspectives

Materials used in cardiovascular surgery must fulfill a few essential requirements: they must be hemostatic, hold sutures, be resistant to pressure and stress while being tissue-friendly and resistant to thrombosis. Additionally, an ideal material has normal tissue function and capability for growth and self-repair.

TE materials can potentially fulfill all of these essential requirements and i*n situ* TE using polymer materials can offer a simplified and potentially cost-effective method to produce off-the-shelf available, TE cardiovascular implants. Although initial results are promising (55), future research is necessitated and there are still many obstacles to overcome before the use of these materials can become a part of the everyday practice of cardiovascular surgery (89). The use of novel cell free techniques to enhance the process of regeneration in TE include adding exosomes (90), hydrogels (91), direct (92, 93), or indirect induced pluripotent stem cell reprogramming using gene editing (94, 95) as well as stimulating myocardial cell division (96). Adding such strategies holds great promise in the future.

## Author Contributions

AD: drafting the first manuscript. MY and JK: critically revising the work for important intellectual content.

### Conflict of Interest

The authors declare that the research was conducted in the absence of any commercial or financial relationships that could be construed as a potential conflict of interest.
